# Effect of platelet-rich fibrin on microperfusion during early socket healing: a randomized controlled clinical trial

**DOI:** 10.1007/s00784-025-06470-7

**Published:** 2025-07-29

**Authors:** Marie Sophie Katz, Mark Ooms, Marius Heitzer, Maurice Klein, Philipp Winnand, Timm Steiner, Frank Hölzle, Ali Modabber

**Affiliations:** Department of Oral and Maxillofacial Surgery, University Hospital RWTH Aachen, Pauwelsstraße 30, 52074 Aachen, Germany

**Keywords:** Platelet-rich fibrin, Ridge preservation, Wound healing, Tooth extraction, Laser-Doppler flowmetry

## Abstract

**Objectives:**

This study aimed to evaluate and compare the early healing of fresh alveolar sockets treated with or without platelet-rich fibrin (PRF) using laser Doppler flowmetry and tissue spectrophotometry (LDF-TS). The primary outcome was gingival perfusion; secondary outcomes included clinical wound healing (based on the Landry Wound Healing Index) and patient-reported postoperative pain.

**Materials and methods:**

Sixty-two patients requiring single tooth extraction were randomized into two groups. In the PRF group, an advanced PRF (A-PRF+) plug was placed in the socket before suturing; in the control group, only suturing was performed. Gingival perfusion was measured at four sites preoperatively and on postoperative days 3 and 10 using LDF-TS. Patients rated pain, and wound healing was clinically assessed. Twelve patients were lost to follow-up, leaving 50 for analysis.

**Results:**

No significant differences were found between the PRF and control group regarding pain (day 3: *p* = 0.654; day 10: *p* = 0.329) or wound healing (day 3: *p* = 0.178; day 10: *p* = 0.595). Perfusion parameters also showed no significant group differences between baseline and day 10: oxygen saturation (SO₂: *p* = 0.884), relative hemoglobin (rHb: *p* = 0.387), and blood flow (*p* = 0.072).

**Conclusions:**

Gingival perfusion showed no significant group differences over 10 days. PRF did not significantly reduce pain or improve wound healing.

**Clinical relevance:**

PRF does not appear to significantly enhance healing, pain reduction, or perfusion in simple extractions. Future studies should use split-mouth designs and focus on more complex surgeries to better evaluate PRF’s effects.

**Trial registration:**

All procedures performed in this study involving human participants were in accordance with the ethical standards of the institutional and/or national research committee and with the 1964 Helsinki Declaration and its later amendments or comparable ethical standards. The study was performed according to the Consolidated Standards of Registered Trial (CONSORT) guidelines. The study was approved by the institutional Clinical Research Ethics Committee (Decision Number 23–105) and by the German Clinical Trials Register (File Number DRKS00032344, registered on October 11, 2023).

## Objectives

Tooth extractions initiate a cascade of biological processes that lead to socket healing through blood clot formation, development of a provisional connective tissue matrix, and subsequent bone maturation and remodeling [1]. Significant dimensional changes of the alveolar ridge occur particularly within the first 8–12 weeks following extraction [2–4]. To counteract these changes and support oral soft and hard tissue healing, various ridge preservation techniques have been developed, involving bone substitute materials, membranes, or platelet concentrates [5–8].

Platelet-rich fibrin (PRF) is a well-established, fully autologous platelet concentrate (APC) that is frequently used to enhance soft and hard tissue healing after oral surgery [9–13]. As a second generation APC, a solid advanced PRF (A-PRF+) plug can be generated using a relatively slow centrifugation protocol, which leads to high releases of growth factors such as VEGF, TGF-β, and EGF during the first week [14–16]. Furthermore, PRF seems to not only influence osteoblasts but also accelerate endothelial growth and promote early vascularization [17]. Various studies have shown that PRF plugs can support open wound healing following dental extractions and may also be used for ridge preservation, as they help stabilize alveolar dimensions during the first three postoperative months postoperatively [7, 18–20]. In addition to its clinical effect on oral regeneration, some studies have reported lower pain associated with PRF use and a lower incidence of alveolar osteitis [21, 22].

While clinical studies evaluating the use of PRF in alveolar wound healing have mainly focused on visible clinically indices, such as the Landry Wound Healing Index (WHI) [18, 23] or the modified WHI by Mozzati et al. [24, 25], early ridge vascularization after dental extractions is difficult to access in vivo, and evidence regarding the effectiveness of PRF in enhancing vascularization and microperfusion following simple extractions remains limited.

Laser Doppler flowmetry (LDF) is a diagnostic tool mostly used to monitor microvascular skin transplants in plastic and maxillofacial surgery, as well as to evaluate vascularization in diabetic ulcers or patients with other malperfusion issues [26–32]. It has also been used to measure hyperperfusion in states of oral inflammation, such as gingivitis or peri-implantitis [33–35], or to track healing after periodontal surgery [36, 37].

Alssum et al. used LDF in a study on single-tooth extractions and observed decreased perfusion in the first postoperative month, which was followed by a phase of hyperemia [38]. In an animal study, Liu et al. evaluated the effect of A-PRF on postoperative alveolar vascularization at the microvascular level by using LDF and showed that A-PRF had a positive impact on angiogenesis in the gingiva of beagle dogs through the induction of VEGF expression [39].

In addition to the classic LDF, which only measures blood flow, LDF and tissue spectrophotometry (LDF-TS) can provide information about the relative amount of hemoglobin (rHb) and oxygen saturation (SO_2_) [29, 33, 34]. These parameters allow researchers a better understanding of the microvascular processes and vascularization of the gingiva. Notably, Mayr et al. showed that lower baseline SO_2_ values could be a warning signal for possible wound healing disorders after oral surgery, which were found especially in smokers with periodontitis [40].

The aim of this study was to evaluate the influence of a solid A-PRF + plug compared to a control procedure (sutures alone) on early healing and vascularization of extraction sockets, as well as on postoperative microperfusion, as measured by LDF-TS. Pain, wound healing, and other postoperative complaints after single tooth extraction were also assessed.

## Methods

### Study design

This randomized clinical trial was approved by the institutional Clinical Research Ethics Committee (Decision Number 23–105) and by the German Clinical Trials Register (File Number DRKS00032344, registered on October 11, 2023). Sixty-two patients requiring a single tooth extraction were randomized into two groups: the experimental group received an advanced platelet-rich fibrin (A-PRF+) plug placed in the extraction socket prior to suturing, while the control group underwent suturing without PRF application. Gingival perfusion was measured preoperatively and on postoperative days 3 and 10 using LDF-TS.

The primary outcome measure was gingival perfusion, secondary outcomes included clinical wound healing assessed by the Landry Wound Healing Index (WHI) and patient-reported postoperative pain levels.

The null hypothesis of this study was that there is no difference between the PRF and control groups regarding gingival perfusion, wound healing, and postoperative pain.

#### Sample size calculation

The existing literature on clinical studies about ridge preservation with PRF and intraoral use of LDF was reviewed to calculate a suitable sample size. The calculation was based on the following: a randomized clinical trial by Alrayyes et al., who evaluated the effect of A-PRF on wound healing after dental extractions in smokers and found enhanced wound closure and healing patterns compared to collagen plugs [18]; a study that used LDF-TS to measure gingival blood flow in patients with and without gingivitis [33]; and a study by Akkaya et al. that measured gingival perfusion using LDF after application of PRF and diode laser on extraction sockets and found a positive effect on early bone regeneration when PRF in combination with diode laser treatment was performed and a significant increase in gingival perfusion during the first week [41].

To calculate the sample size, differences in blood flow values were defined as the primary outcome and the WHI as the secondary outcome. The statistical program G* Power Version 3.1.9.6 (Heinrich-Heine-Universität, Düsseldorf, Germany) was used for the calculation, with an alpha value of 0.05, an effect size of 0.5, and a statistical power of 90%. Based on these parameters, a sample size of at least 47 patients (23.5 per group) was calculated to achieve 95% power and a 95% confidence interval for detecting differences in perfusion between the groups. To account for potential dropouts, we aimed to include at least 50 patients (25 per group). Therefore, a total of 62 patients were initially enrolled. After 12 dropouts, the final sample consisted of 50 patients, as planned.

#### Eligibility Criteria

All participants were recruited in the Department of Oral and Maxillofacial Surgery, University Hospital RWTH Aachen between August 2023 and May 2025. To be included in this study, patients had to be at least 18 years of age, in good general health, and in need of a single tooth extraction. Patients could only be included if they consented to participate by signing a written informed consent form. Further exclusion criteria were signs of an acute infection or swelling before the operation, pregnancy, bleeding disorders, medications such as anticoagulants or other medication that could affect perfusion or wound healing, and patients who had smoked less than two hours before the examination. All patients agreed not to smoke for at least 10 days after the operation.

#### Randomization

The participants were enrolled by one researcher (MSK), who monitored the study’s execution. The participants were informed about the randomization but did not know until the day of the operation whether or not an A– PRF + plug would be inserted into the socket. A simple randomization method without restrictions was used. Prior to the operation, the surgeon drew a sealed envelope that had been randomly mixed from a set of 50 (25 containing a “0” and 25 a “1”). A “1” indicated that an A-PRF + plug would be applied, while a “0” indicated that the wound would be closed using regular suturing only (control group). The numbers were not visible from the outside, and the envelopes were indistinguishable in size and appearance to ensure allocation concealment. If a patient was excluded due to loss to follow-up, the previously drawn envelope was returned to the pool to maintain the 1:1 group allocation ratio throughout the study.

After data collection, another surgeon (MO) conducted the data analysis. To eliminate bias, the data were pseudonymized before the analysis.

#### Blinding

The study was not blinded due to the nature of the intervention. Both the surgeon and the patient were aware of the treatment allocation at the time of surgery.

### Surgical procedure

Each patient underwent a regular dental extraction under local anesthesia in the same operating theater. Preoperatively, patients were asked about their current pain on a visual analogue scale (VAS) and rinsed their mouths with an antibacterial chlorhexidine mouthwash. In cases where simple extraction was not possible, a marginal incision, root separation (for multirooted teeth, especially molars), and osteotomy were performed and documented.

**Fig. 1:**
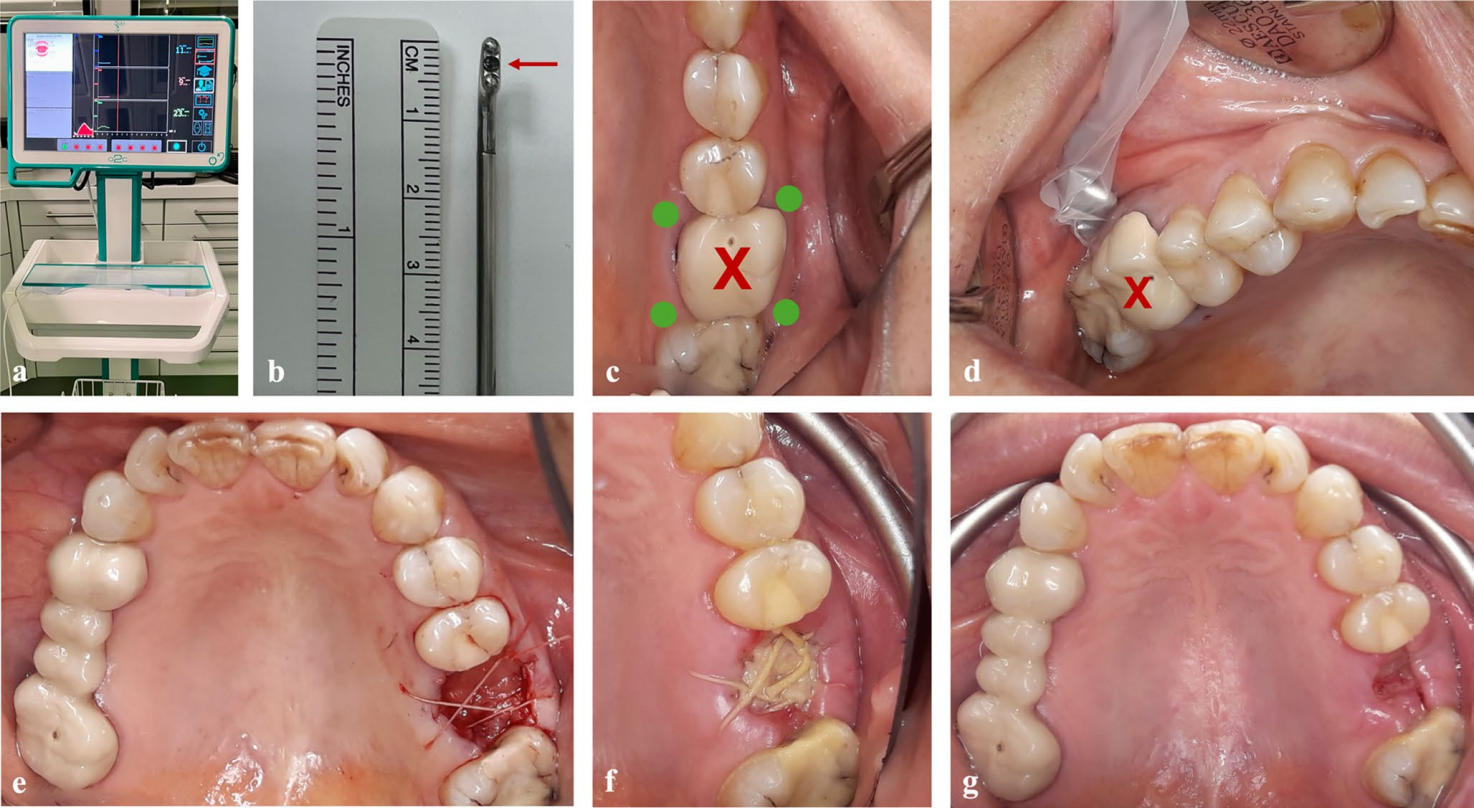
Perfusion measurements before and after ridge preservation with an A-PRF + plug. (**a**) Oxygen to see (O2C) device; (**b**) gingival probe, arrow indicating the measuring point; (**c**) measurement points adjacent to the non-restorable tooth (X); (**d**) gingival measurement preoperatively; (**e**) the socket was filled with an A-PRF + plug after extraction and sutured; (**f**) clinical assessment and gingival measurement on postoperative day 3; (**g**) clinical assessment, gingival measurement, and removal of the sutures on postoperative day 10

In the PRF group, an A-PRF + plug was inserted into the socket, and adapted sutures were placed above it (Ethicon Vicryl^®^ 4 − 0, Somerville, NJ, USA) (Fig. 1e). In the control group, the extraction socket was sutured in the same manner but without the insertion of an A-PRF + plug.

Postoperatively, patients received standardized instructions regarding oral hygiene, cooling, smoking cessation, and were advised to take 600 mg ibuprofen every 6–8 h as needed for pain and inflammation for the first 3 days.

### Preparation and application of the A-PRF + plug

If patients were allocated to the PRF group, prior to the operation, 20 ml of peripheral venous blood was drawn with a butterfly needle into an A-PRF + vacuum glass tube (Process for PRF, Nice, France). Afterwards, the blood was immediately centrifugated using a PRF-Duo Quattro medical device centrifuge (Process for PRF, Nice, France) at 1300 rpm for 8 min (A-PRF + protocol).

Subsequently, the tubes were left to rest for 10 min, and then the A-PRF + clots were carefully taken out with a sterile tweezer and scissor. The bottom red phase of erythrocytes was removed, and the remaining solid A-PRF + clots were then pressed into two plugs (PRF-Box, Process for PRF, Nice, France). These A-PRF + plugs were then both applied into the socket directly before closing the wound with adaptive sutures (Ethicon Vicryl^®^ 4 − 0, Somerville, NJ, USA).

### Perfusion measurement

All non-restorable teeth were measured using LDF-TS before the operation, prior to the application of local anesthesia to obtain baseline perfusion data. All patients were measured and examined by the same experienced surgeon.

The oxygen to see (O2C) device (LEA-Medizintechnik, Gießen, Germany) used in this study has a gingival probe with a 5 × 2 mm dimension and a predefined measurement depth of 1 mm (LSX-41 gingival probe, LEA-Medizintechnik, Gießen, Germany) (Fig. 1a-b). The patient lay in a comfortable position, and any light except for the main room light was turned off to avoid interference in the measurements. The probe was gently placed on four points adjacent to the non-restorable tooth (buccal mesially, buccal distally, palatal/lingual mesially, and palatal/lingual distally) to attain a representative arithmetic mean (Fig. 1c–d).

Each perfusion measurement took 10 s, and the examiner had 5 s prior to adjust the position of the probe head before recording. A visual control ensured that the examiner was aware of micro-movements and could repeat the measurement if necessary.

### Clinical evaluation and follow-up

All patients were followed up on postoperative day 3 and 10 (Fig. 1f–g). They were asked to evaluate their pain on a visual analogue scale (VAS) ranging from 0 (no pain) to 10 (severe pain), and after the perfusion measurements were completed, the patients were clinically examined.

The wound was assessed using the Landry WHI, which evaluates tissue color, bleeding on palpation, presence of granulation tissue, and epithelialization of the incision margin by rating them on a scale from 1 to 5 (1 = very poor, 2 = poor, 3 = good, 4 = very good, and 5 = excellent) [23].

The sutures were removed on day 10. If the patients showed signs and symptoms of an alveolar osteitis, it was treated by rinsing with saline solution and antibiotics if needed. Subsequently, the perfusion and clinical data were combined, and correlations were evaluated.

### Statistical analysis

For the descriptive statistics of the categorical data (sex, reason for extraction, number of roots, type of tooth removal, smoking habit, location of surgery, incidence of alveolar osteitis), the differences between the groups were analyzed using a Chi-square test, the Fisher-Freeman-Halton test, or Fisher’s test.

The continuous data (age, pain measured on the VAS, the Landry WHI, changes in SO_2_, rHb, and blood flow) were evaluated using the Mann–Whitney test, as they lacked a Gaussian distribution according to the Shapiro–Wilk test. P-values < 0.05 were considered significant. Testing for differences in changes in median SO_2_, median rHb, and median blood flow between the groups, we controlled for sex, age, removal technique, number of roots, and smoking habit using linear regression analysis.

## Results

A total of 62 patients (aged 18–85 years) requiring a single tooth extraction were informed about the study objectives and design, consented to participate, and signed a written informed consent form. Twelve patients were lost to follow-up (five from the PRF group and seven from the control group). These dropouts occurred either before the 3-day or the 10-day follow-up visit, resulting in incomplete postoperative data for these individuals. The final study population thus included 50 patients, with 25 treated with PRF and 25 in the control group (Table 1).

**Table 1:** Patient cohort and group distribution

	Control group	PRF^a^ group	Total	*P*-value
Sex				
**Male**	9 (36%)	14 (56%)	23 (46%)	0.156
**Female**	16 (64%)	11 (44%)	27 (54%)	
**Median age (in years)**				
	51 (IQR ± 27.5)	49 (IQR ± 22)	50 (IQR ± 22.25)	0.900
**Reason for extraction**				
**Profound caries**	13 (52%)	11 (44%)	24 (48%)	0.304
**Chronic periapical lesion**	5 (20%)	7 (28%)	12 (24%)	
**Periodontal disease**	3 (12%)	6 (24%)	9 (18%)	
**Orthodontic treatment**	2 (8%)	0 (0%)	2 (4%)	
**Vertical fracture**	2 (8%)	0 (0%)	2 (4%)	
**Root resorption**	0 (0%)	1 (4%)	1 (2%)	
**Location**				
**Maxilla**	12 (48%)	14 (56%)	26 (52%)	0.571
**Mandible**	13 (52%)	11 (44%)	24 (48%)	
**Number of roots**				
**1**	5 (20%)	4 (16%)	9 (18%)	0.924
**2**	10 (40%)	11 (44%)	21 (42%)	
**3**	10 (40%)	10 (40%)	20 (40%)	
**Type of removal**				
**Extraction**	21 (84%)	20 (80%)	41 (82%)	0.725
**Osteotomy**	4 (16%)	5 (20%)	9 (18%)	
**Smoking habit**				
**Yes**	4 (16%)	5 (20%)	9 (18%)	1.000
**No**	21 (84%)	20 (80%)	41 (82%)	

^a^PRF: platelet-rich fibrin; ^b^VAS: visual analogue scale

The median age was 50 (interquartile range [IQR] ± 22.25) years: control group median age 51 [IQR ± 27.5] years and PRF group median age 49 [IQR ± 22] years. Twenty-three procedures were performed on male patients (46%), and 27 dental extractions were carried out on female patients (54%). There was no significant difference in age (*p* = 0.900) or sex (*p* = 0.156) between the control and PRF groups.

The most common reason for extraction was profound caries (48%), followed by chronic periapical lesion (24%), periodontal disease (18%), orthodontic treatment (4%), vertical tooth fractures (4%), and root resorption (2%). The distribution of reasons for the procedure was also not statistically different between the groups (*p* = 0.304). Twenty-six procedures were carried out in the maxilla (52%) and 24 in the mandible (48%), equally distributed between the PRF and control groups (*p* = 0.571).

Most teeth had two (42%) or three (40%) roots and could be removed by simple extraction (82%). There was also no significant difference between the control and PRF groups regarding the number of roots (*p* = 0.924) or the distribution of extractions and osteotomies (*p* = 0.725).

Nine patients had a history of regular smoking but testified that they had not smoked or least not during the two hours before and ten days after the operation (five in the PRF group, and four in the control group, *p* = 1.000). The median pain on the VAS before the operation was 0, and there was no significant difference between the groups (*p* = 0.602).

**Fig. 2:**
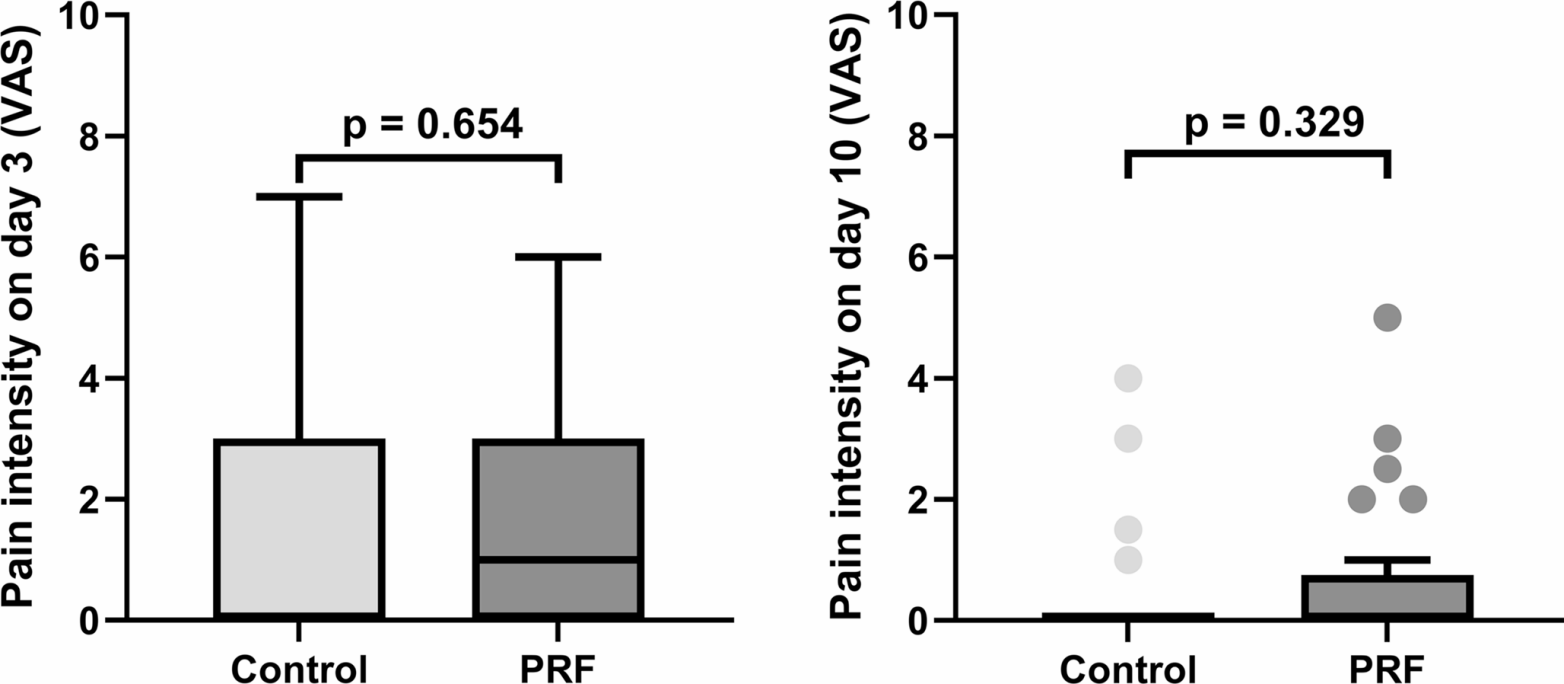
Differences in pain on the visual analogue scale (VAS) between the control and platelet-rich fibrin (PRF) groups on postoperative day 3 (**a**) and on postoperative day 10 (**b**). Each bar represents the interquartile range (IQR), with the top of the bar indicating the upper quartile. The horizontal lines correspond to the medians of the respective groups (Control group: day 3 and day 10 = 0; PRF group: day 3 = 1, day 10 = 0). The whiskers indicate lower and upper extremes, excluding outliers

When comparing postoperative pain, which was low overall (day 3: median VAS_Control_ = 0; median VAS_PRF_ = 1; day 10: median VAS_Control_ = 0; median VAS_PRF_ = 0), there was no significant difference between the groups on postoperative days 3 or 10 (*p* = 0.654, and *p* = 0.329, respectively) (Fig. 2).

**Fig. 3:**
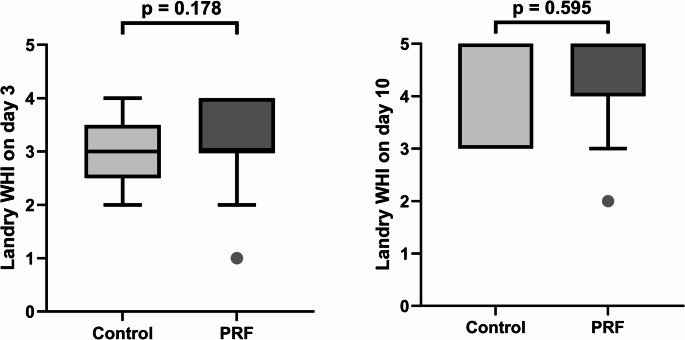
Differences in the Landry Wound Healing Index (WHI) between the control and platelet-rich fibrin (PRF) groups on postoperative day 3 (**a**) and on postoperative day 10 (**b**). Each bar represents the interquartile range (IQR), with the top indicating the upper quartile and the bottom the lower quartile. The horizontal lines indicate the medians for each group (Control group: day 3 = 3, day 10 = 5; PRF group: day 3 = 3, day 10 = 5). The whiskers indicate lower and upper extremes, excluding outliers

The Landry WHI also showed favorable results in both groups (day 3: median WHI_Control_ = 3; median WHI_PRF_ = 3; day 10: mean WHI_Control_ = 5; mean WHI_PRF_ = 5), and there was no significant difference between the groups on day 3 or day 10 (*p* = 0.178, and *p* = 0.595, respectively) (Fig. 3).

Four patients developed symptoms of an alveolar osteitis (two in each group), indicating no significant difference between the control and PRF groups (*p* = 1.000). Similarly, no statistically significant differences were observed in the incidence of an alveolar osteitis with respect to smoking habits (*p* = 1.000), sex (*p* = 0.614), indications for tooth removal (*p* = 1.000), location (maxilla vs. mandible; *p* = 0.340), or number of roots (*p* = 0.507). Although alveolar osteitis occurred in 22.2% of all osteotomies and only 4.9% of all simple extractions, this difference was not statistically significant (*p* = 0.174).

**Fig. 4:**
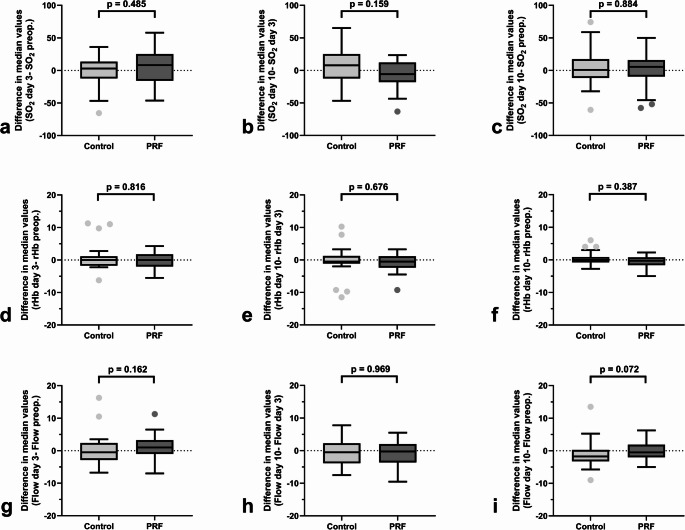
Differences in perfusion values between postoperative day 3 and preoperative (baseline) values (**a**, **d**, **g**); between postoperative day 10 and postoperative day 3 (**b**, **e**, **h**); and postoperative day 10 and preoperative (baseline) values (**c**, **f**, **i**). Bars show the median values (SO_2_, rHb, and blood flow); top and bottom of the boxes show the lower and upper quartiles; whiskers show lower and upper extremes, excluding outliers

The difference between the median oxygen saturation SO_2_ (%), median relative amount of hemoglobin rHb (AU), and median blood flow (AU) did not differ between the groups on postoperative day 3 compared to the preoperative baseline values (*p* = 0.485, *p* = 0.816, and *p* = 0.162, respectively) (Fig. 4a, d, g). Nor did the values differ between the groups when the day 3 and day 10 perfusion measurements were compared (*p* = 0.159 for SO_2_, *p* = 0.676 for rHb, and *p* = 0.969 for blood flow, respectively) (Fig. 4b, e, h). Similarly, when comparing the difference between the postoperative day 10 and the preoperative baseline values, SO_2_, rHb, and blood flow did not differ significantly (*p* = 0.884, *p* = 0.387, and *p* = 0.072, respectively) (Fig. 4c, f, i).

When controlling for sex (*p* = 0.108), age (*p* = 0.196), removal technique (*p* = 0.391), number of roots (*p* = 0.699), and smoking habit (*p* = 0.650) through regression analysis, the differences in median SO_2_ changes between the postoperative day 10 and preoperative values in the PRF and control groups remained statistically nonsignificant different (*p* = 0.441) (Table 2).

**Table 2:** Regression analysis to test the differences in median SO_2_ changes (%) between day 10 and baseline values in patients with and without PRF

Parameter	Value	ß	*P*- value
PRF	No vs. Yes	−6.124	0.441
Sex	Male vs. Female	−13.409	0.108
Age	(In years)	−0.319	0.196
Removal technique	Extraction vs. Osteotomy	−8.896	0.391
Number of roots	Single vs. two or three roots	−4.044	0.699
Smoking habit	Non-smokers vs. Smokers	4.869	0.650

When performing regression analysis for changes in median rHb values, and controlling for sex (*p* = 0.563), age (*p* = 0.153), removal technique (*p* = 0.857), number of roots (*p* = 0.893), and smoking habit (*p* = 0.114) through regression analysis, the differences in median rHb changes between the day 10 and preoperative values in the PRF and control groups remained statistically nonsignificant (*p* = 0. 927) (Table 3).

**Table 3:** Regression analysis to test the differences in median rHb changes (AU) between postoperative day 10 and baseline values in patients with and without PRF

Parameter	Value	ß	*P*- value
PRF	No vs. Yes	0.136	0.927
Sex	Male vs. Female	−0.887	0.563
Age	(In years)	−0.066	0.153
Removal technique	Extraction vs. Osteotomy	−0.347	0.857
Number of roots	Single vs. two or three roots	0.262	0.893
Smoking habit	Non-smokers vs. Smokers	3.200	0.114

Additionally, when controlling for sex (*p* = 0.280), age (*p* = 0.191), removal technique (*p* = 0.732), number of roots (*p* = 0.720), and smoking habit (*p* = 0.174) through regression analysis, the differences in median blood flow changes between the postoperative day 10 and preoperative values of the PRF and the control groups remained statistically nonsignificant (*p* = 0.479) (Table 4).

**Table 4:** Regression analysis to test the differences in median blood flow changes (AU) between postoperative day 10 and baseline values in patients with and without PRF

Parameter	Value	ß	*P*- value
PRF	No vs. Yes	4.926	0.479
Sex	Male vs. Female	−7.832	0.280
Age	(In years)	−0.282	0.191
Removal technique	Extraction vs. Osteotomy	−3.096	0.732
Number of roots	Single vs. two or three roots	3.281	0.720
Smoking habit	Non-smokers vs. Smokers	12.869	0.174

## Discussion

PRF is frequently used for ridge preservation and activation of biomaterials and as a hemostatic plug after dental extractions [19, 42–44]. Previous studies have demonstrated that PRF can accelerate wound healing after tooth extraction, stabilize alveolar ridge dimensions during early bone healing, and reduce postoperative pain [19, 45, 46]. However, our study did not find significant differences between the PRF and control groups regarding gingival perfusion, wound healing and postoperative pain. Accordingly, the null hypothesis—that there is no difference between the groups in these outcomes—was accepted.

Overall, postoperative pain levels were considerably low in this study population, with a median value of 0 (control group) and 1 (PRF group) on day 3 and a median value of 0 (both groups) on day 10. This may be attributed to the fact that only 18% of the procedures involved osteotomies, and the wound size from single tooth extraction was relatively small. Similarly, Ustaoğlu et al., compared PRF use after single tooth extractions to natural socket healing and found significant pain reduction only on the first postoperative day, with no difference on days two and three [11]. Gatti et al., who evaluated pain after free gingival graft harvesting and treatment with L-PRF compared to cellulose, also found no significant differences regarding pain or soft tissue healing [47].

Conversely, Iftikhar et al. observed a significant benefit of PRF in reducing pain after impacted third molar surgery [48]. These findings suggest that the relatively low pain levels following single tooth extractions may limit the ability to detect the superiority of PRF in pain reduction.

The randomized controlled design with sealed envelope allocation ensured a robust method for treatment assignment and minimized selection bias. Performing all procedures in the same operating theater under standardized conditions further contributed to consistency. However, this study was not blinded, as both surgeons and patients were aware of the treatment allocation, which may have introduced performance or detection bias, particularly in subjective outcomes such as pain assessment.

We included only healthy patients with a good wound healing capacity, which explains the excellent Landry WHI scores on day 3 (control group: 3; PRF group: 3), as well as on day 10 (control group: 5; PRF group: 5).

The differences in VAS scores after simple single tooth extraction were not significant. These results align with Ustaoğlu et al., who found no significant differences between socket healing with and without PRF using the Landry WHI; however, they still found better epithelization rates in the PRF groups [11]. Giudice et al., also using an A-PRF + protocol, observed less postoperative bleeding with A-PRF + compared to suturing alone, but also no significant difference in wound healing when comparing PRF plugs with a hemostatic plug or suturing alone [49]. Nonetheless, PRF might benefit patients with compromised wound healing, as shown in studies on patients with type 2 diabetes [50], or those receiving antiresorptive therapy [51].

In this study, four cases of alveolar osteitis (dry sockets) occurred, showing no correlation with PRF use. While alveolar osteitis seems to be more common in females, molar extractions, and in procedures involving osteotomies, no significant differences were observed in our study, likely due to the small number of osteotomies included [52, 53]. Previous studies have suggested PRF may stabilize blood clots and prevent alveolitis in dental extractions and third molar surgery [21, 54], but given the rarity of this complication in simple extractions, larger cohorts are needed to evaluate this effect [46, 55].

The Landry WHI is widely used for assessing alveolar healing, but a review by Rodriguez et al. highlighted its subjectivity due to lack of quantitative and objective subclinical parameters relying solely on visual clinical appearance [56]. To address this potential bias clinical evaluations were complemented by perfusion measurements, allowing better insight into the microvascular dynamics.

Nevertheless, several limitations should be considered when interpreting our findings. The sample size was relatively small, and twelve patients were lost to follow-up, possibly reducing statistical power to detect subtle differences. Furthermore, the study was not blinded; both surgeons and patients were aware of treatment allocation, potentially introducing bias, especially in subjective outcomes such as pain assessment. Finally, the inclusion of only healthy patients with good healing capacity limits the generalizability of the results to populations with compromised wound healing.

Liu et al. demonstrated that PRF induces VEGF expression and promotes angiogenesis in gingiva of beagle dogs [39]. In contrast, we observed no significant difference in blood flow between the groups in humans. This discrepancy may relate to methodological differences: Liu et al. used an LDF probe with 2 mm measurement depth, while the probe used in this study measured 1 mm deep, possibly missing peripheral vascularization changes during the first 10 days [39].

Moreover, as we previously showed in a peri-implant perfusion study involving patients with microvascular reconstruction, inter-individual perfusion can show considerable variation [34], complicating interpretation across different patients. This limitation might be addressed in future split-mouth studies that control for patient-specific variables.

Alssum et al. described an ischemia-reperfusion model following single tooth extractions in a study involving 15 healthy patients [38]. While we observed no significant differences in blood flow from baseline to day 3 or 10 postoperatively, the PRF group showed a slight increase in perfusion on day 3 (+ 1.00 AU median difference), whereas the control group showed a slight decrease (–0.50 AU median difference).

As the initial postoperative period is primarily characterized by an inflammation, this may explain the transient increased gingival blood flow [4]. Subsequently, both groups showed a gradual decrease in blood flow by day 10. In contrast, Akkaya et al. reported a continuous increase in gingival perfusion during the first postoperative week, as measured by LDF [41]. Although PRF positively influenced early bone regeneration in their study, it did not have a significant effect on gingival perfusion, aligning with our findings [41].

Besides PRF, other biologics such as platelet-rich plasma (PRP), concentrated growth factors (CGF), and hyaluronic acid (HA) have been explored for alveolar ridge preservation. PRP, a first-generation platelet concentrate, appears to contain lower levels of growth factors compared to PRF or CGF [57]. A study by Yelamali et al. demonstrated that PRF significantly outperforms PRP in promoting soft tissue healing and accelerating bone regeneration 4 months after removal of third molars [58]. CGF is considered a third-generation platelet concentrate. According to Huang et al. and Al-Badran et al., CGF may reduce postoperative pain and enhance soft tissue healing [59, 60]. However, another study by Iao et al. suggests that “sticky bone” prepared with A-PRF releases significantly more growth factors than CGF-based sticky bone [61]. To date, only a limited number of studies have directly compared the effects of CGF and PRF on oral wound healing [61–63].

Hyaluronic acid has also been studied for its potential to enhance wound healing and bone regeneration, either as a standalone agent or in combination with xenogenic bone substitutes [64, 65]. While some studies report beneficial outcomes, others have found no significant effect [64–68]. Overall, the available evidence supporting the clinical use of HA in this context remains considerably weaker than that for PRF [69, 70].

## Conclusions

In this study, both the PRF and control groups showed similarly low pain levels, favorable healing, and comparable gingival perfusion values during the first 10 postoperative days, with no statistically significant differences observed between groups. The effect of PRF may be more pronounced in more invasive procedures such as osteotomies or third molar surgeries. Future studies investigating microperfusion with and without PRF should consider a split-mouth design and focus on more invasive surgical approaches to minimize inter-individual variability and better assess potential benefits.

## Data Availability

The data that support the findings of this study are available from the corresponding author upon reasonable request.
